# Characteristic and Synthesis of High-Temperature Resistant Liquid Crystal Epoxy Resin Containing Boron Nitride Composite

**DOI:** 10.3390/polym14061252

**Published:** 2022-03-20

**Authors:** Li-Chuan Wu, Yi-Wen Huang, Yao-Ming Yeh, Chih-Hung Lin

**Affiliations:** 1 Department of Applied Chemistry and Material Sciences, Fooyin University, 151 Jinxue Road, Daliao, Kaohsiung City 83102, Taiwan; sc023@fy.edu.tw; 2 Department of Applied Chemisrty, National Yang Ming Chiao Tung University, 1001 University Road, Hsinchu 30000, Taiwan; yiwen.nl07g@nctu.edu.tw (Y.-W.H.); fightingbird.ac05g@nctu.edu.tw (Y.-M.Y.); 3 Center for General Education, Chang Gung University of Science and Technology, 261 Wen-Hwa 1st Road, Kwei-Shan, Tao-Yuan 33303, Taiwan

**Keywords:** thermal conductivity, liquid crystalline epoxy resin, boron nitride composite

## Abstract

Five liquid crystal epoxy resins and composites containing flat boron nitride (f-BN) and spherical boron nitride (s-BN) were successfully synthesized. The chemical structures, crystal diffraction, and thermal conductivity of the liquid crystal (LC) epoxy composites were measured using Nuclear Magnetic Resonance (NMR), Differential Scanning Calorimetry (DSC), X-ray, and Discovery Xenon Flash. In this study, the molecular arrangement of five LC epoxy resins and the thermal conductivity of their composites were carefully discussed. Several different amounts of flat boron nitride and spherical boron nitride were added to the five LC epoxy resins. The influence of nano-scale ceramic materials, f-BN, and s-BN, on the thermal conductivity of the LC epoxy resins, was studied. It is worth noting that the thermal conductivity of the spherical boron nitride composite demonstrated a better result than that of the flat boron nitride composite. In simpler terms, the thermal conductivity of the composites is closely related to the molecular arrangement of the LC resin and the amount of BN added. The results demonstrate that the SBPDAE/s-BN (60%) composite shows the highest thermal conductivity of 9.36 W/mK in the vertical direction. These data prove that the LC alignment of the matrix will greatly enhance the thermal conductivity of the composites.

## 1. Introduction

Liquid crystals are intermediate in symmetry and structure between the solid crystalline state and the amorphous liquid state. In the temperature of liquid crystalline mesophase, liquid crystal polymers (LCPs) have a low viscosity, and the molecules can be arranged in a highly self-aligned direction along the flow direction. Due to the rigid structural characteristics of the liquid crystal molecules, LCP materials show a series of excellent properties, such as high strength, high modulus, high heat resistance, flame resistance, high dimensional stability, high fluidity, excellent electrical insulation, chemical resistance, self-reinforcing effect, weathering resistance, microwave penetration, and excellent processing performance [1,2]. According to the thermal deformation behavior of LCPs, they can be divided into three categories: type I (above 250 °C), type II (180–250 °C), and type III (100–200 °C). Type I LCPs are difficult to process due to their high-temperature range. In contrast, the temperature range of type III LCPs is too low, which leads to their limited application. Therefore, type II LCPs are the most widely used in the industry. Among type II LCPs, a polymer composed of 4-hydroxybenzoic acid (HBA)/2-hydroxy-6-naphthoic acid (HNA) as the main chain structure is the most famous one.

Difunctional LC monomers have been widely studied in the literature because these kind of reactive LC monomers can be easily aligned and polymerized to form oriented LC networks. During polymerization, the ordering of the mesogen was fixed, yielding a uniaxially oriented polymer network. This kind of optical anisotropic LC network can be used as an optical compensation film for an LC display to resolve its narrow-viewing angle problem [3,4,5,6,7]. CPs are mainly used as electronic connector materials, packaging materials, fiber materials, medical equipment, etc. In recent years, the line width of integrated circuits has become smaller and, following Moore’s law, electronic components are developing towards integration, miniaturization, and functionalization. Heat dissipation, therefore, becomes a serious problem when the circuit design is downsizing. As a result, the demand for high thermal conductivity electronic packaging materials is increasing. However, the heat conduction characteristic mainly lies in the materials. In terms of metal materials, the heat conduction mainly depends on free electrons, so the thermal conductivity is quite high. On the other hand, inorganic ceramics and polymer materials mainly rely on lattice vibration only. Most polymers have an amorphous characteristic, and their thermal conductivities are usually between 0.1~0.6 W/mK. The character of inorganic ceramics is relatively higher; their thermal conductivity is typically 2–3 orders of magnitude higher than that of polymers [8] There are several ways to improve the thermal conductivity of polymer materials. One effective method is to add high thermal conductivity inorganic fillers, such as AlN, Al_2_O_3_, Si_3_N_4_ [9,10,11,12,13], BN [14,15,16,17,18,19,20,21], etc. The thermal conductivity of single-layer carbon nanotubes could reach 3000W/mK, and that of graphene can be even higher, reaching 5000W/mK.

In view of the application of liquid crystal materials with high thermal conductivity, we synthesized 4,4′-Biphenol diglycidyl ether (BPE), and three other new liquid crystal epoxy resins (LCERs), 4,4′-bis(4-hydroxybenzylidene)Diaminophenylene Diglycidyl Ether (SBPDAE), 1,4-Bis{[4-(2,3-epoxypropoxy)phenyliminomethylidene]-benzoyloxyl}butane (SBBDAE), and 1,8-Bis{[4-(2,3-epoxypropoxy) phenyliminomethylidene]-benzoyloxyl}octane (SBODAE). The newly synthesized LCERs would be expected to exhibit a melting point of about 180~250℃, and therefore can be suitable for the application in packing materials. Different kinds of boron nitride were chosen to add into the resins for studying the thermal conductivity of the composites [22,23,24,25,26,27,28,29,30,31].

## 2. Materials and Methods

### 2.1. Experimental Reagents

All chemical agents and solvents were purchased from Acros Organics (Geel, Belgium), Alfa Aesar (Ward Hill, MA, USA), Sigma (Ronkonkoma, NY, USA), Aaron Chemicals (USA), and Showa Chemical (Tokyo, Japan).

### 2.2. Instruments

^1^H-NMR spectra were measured using a Bruker Am 400 Spectrometer with tetramethylsilane (TMS) as an internal standard and CDCl_3_ or DMSO-D_6_ as a solvent. The thermal characterization was measured with a TA Instruments Q200 differential scanning calorimeter (DSC). In-site multi-functional X-ray diffraction by Bruker APEX DUO was used for obtaining single-crystal diffraction patterns. TA Discovery Xenon Flash DXF 200 was used for measuring thermal diffusivity, thermal conductivity. The fracture surfaces of the specimens were investigated using field emission scanning electron microscopy (JEOL JSM-7401F FE-SEM) at an accelerating voltage of 20 kV.

### 2.3. Synthesis of Liquid Crystal Epoxy Resin

**Scheme 1:**
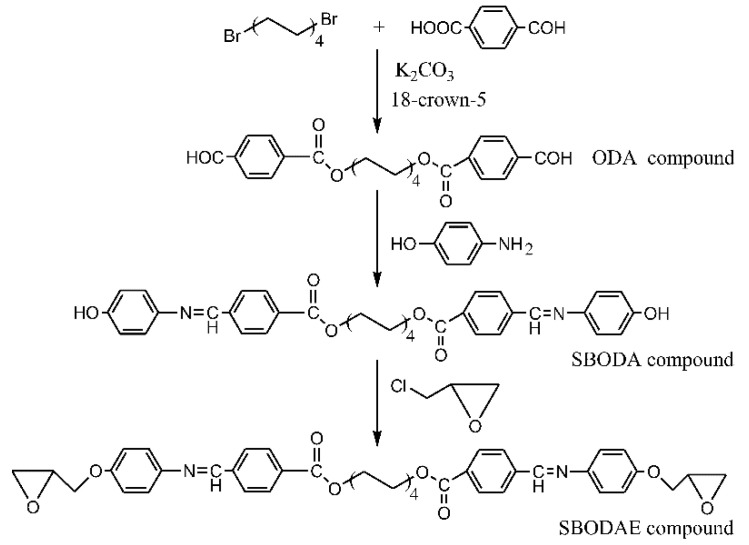
Synthesis routes of target compounds.

All the four target compounds, viz. BPE, SBPDAE, SBBDAE, and SBODAE were prepared by a similar method. The precursor (epoxide) synthetic route is well known [32,33]. Taking compound SBODAE as an example, the synthetic routes of intermediates and target compounds were represented in Scheme 1. TLC and ^1^H-NMR spectroscopy were used to verify the chemical structures and the purity of the intermediates and target compounds. The synthesis methods and analysis of each product are described below.

#### 2.3.1. 4,4′-Biphenol Diglycidyl Ether (BPE)

4,4′-Biphenol (20 g, 0.107 mol), benzyltrimethylammonium chloride (0.58 g, 0.0031 mol), and epichlorohydrin (174.6 mL, 2.22 mol) were placed in a double-necked round- bottomed flask, and the mixture was stirred and heated to reflux for 30 min under nitrogen atmosphere. A 4.0M sodium hydroxide aqueous solution (53.79 mL) was then slowly dropped into the solution. The solution was continuously stirred and heated to reflux for 1 h. After the reaction had gone to completion, it was cooled to room temperature. The solution appeared a white solid precipitate. The crude product was washed with water and ether and filtered under vacuum. The white solid was then placed in a vacuum oven at 70 °C for drying to obtain a white powder BPE (19.04 g, 59.4%).

^1^H NMR (400 MHz, CDCl_3_, ppm): δ = 7.448–7.483 (d, 4H), δ = 6.954–6.984 (d, 4H), δ = 4.237–4.273 (dd, 2H), δ = 3.982–4.009 (dd, 2H),δ = 3.359–3.398 (m, 2H), δ = 2.912–2.934 (dd, 2H), δ = 2.769–2.789 (dd, 2H).

#### 2.3.2. 4,4′-Bis(4-hydroxybenzylidene)diaminophenylene (SBPDA)

P-phenylenediamine (10 g, 0.0926 mol) and 4-hydroxybenzaldehyde (24.8 g, 0.203 mol) were placed in a double-necked round-bottomed flask equipped with a Dean-Stark distiller. Anhydrous Toluene (250 mL) was slowly added into the mixture, and the solution was stirred and heated to reflux for 18 h under nitrogen atmosphere. The solution was then cooled to room temperature, and a yellow solid was precipitated and filtered under vacuum. The yellow crude product was placed in a vacuum oven at 100 °C for drying. Recrystallization from dimethylformamide (DMF)/water (1/1) afforded a light yellow solid precipitate, followed by drying in a vacuum oven at 70 °C to obtain light yellow powder SBPDA (9.67 g, 33.1%).

^1^H NMR (400 MHz, DMSO-d_6_, ppm): δ = 10.143 (s, 2H), δ = 8.493 (s, 2H), δ = 7.771–7.779 (d, 4H), δ = 7.234–7.250 (d, 4H), δ = 6.871 (s, 4H).

#### 2.3.3. 4,4′-Bis(4-hydroxybenzylidene)diaminophenylene Diglycidyl Ether (SBPDAE)

SBPDA (9.67 g, 0.0306 mol) and tetrabutylammonium bromide (1.01 g, 0.00306 mol) were placed in a double-necked round-bottomed flask, and the mixture was slowly dissolved by N,N-Dimethylacetamide (DMAc) (95.62 mL) under nitrogen. After the addition of epichlorohydrin (118.45 mL, 1.51 mol), the solution was stirred and heated to reflux for 2 h. It was then cooled to room temperature and a yellow solid precipitate appeared. The crude product was washed with water and ether, and then filtered by suction filter. Using a vacuum oven for drying the yellow solid at 70 °C to obtain a yellowish powder SBPDAE (9.40 g, 72.5%).

**Figure 1:**
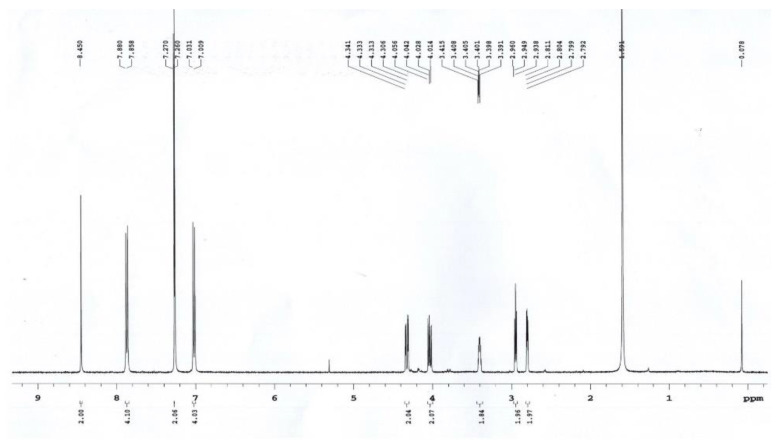
NMR spectra of SBPDAE.

^1^H NMR (400 MHz, CDCl_3_, ppm): δ = 8.450 (s, 2H), δ = 7.858–7.880 (d, 4H), δ = 7.260–7.270 (d, 4H), δ = 7.009–7.031 (s, 4H), δ = 4.306–4.331 (dd, 2H), δ = 4.014–4.056 (dd, 2H), δ = 3.391–3.415 (m, 2H), δ = 2.938–2.960 (dd, 2H), δ = 2.792–2.811 (dd, 2H). The NMR spectra shows in Figure 1.

#### 2.3.4. 1,4-Bis(4-formylbenzoyloxy)butane (BDA)

1,4-dibromobutane (10 g, 0.0463 mol), 4-formylbenzoic acid (27.8 g, 0.185 mol), K_2_CO_3_ (64.01 g, 0.463 mol) and 18-crown-6 (12.24 g, 0.0463 mol) were placed in a double-necked round-bottomed flask. The mixture was dissolved by DMF (250 mL) under nitrogen, and the solution was stirred and heated to 90 °C for 8 h. After the reaction had gone to completion, the solution was cooled to room temperature, and the K_2_CO_3_ was filtered from the reaction solution. A light coffee-colored clear filtrate was obtained. The filtrate solution was poured into ice water and a white solid was precipitated. The mixture was filtered under vacuum to obtain the white solid. The crude product was dissolved in dichloromethane (DCM) and extracted by 10% potassium hydroxide aqueous solution three times. After the extraction, the organic layer was collected and dried over anhydrous MgSO_4_. The solution was then concentrated and distilled to obtain a white solid, followed by drying in a vacuum oven at 70 °C to afford the white powder BDA (9.64 g, 58.8%).

^1^H NMR (400MHz, CDCl_3_, ppm): δ = 10.102 (s, 2H), δ = 8.184–8.203 (d, 4H), δ = 7.940–7.961 (d, 4H), δ = 4.441–4.461 (t, 4H), δ = 1.974–1.993(m, 4H).

#### 2.3.5. 1,4-Bis{[4-(4-hydroxy)phenyliminomethylidene]-benzoyloxyl}butane (SBBDA)

BDA (9.64 g, 0.027 mol) and 4-aminophenol (5.94 g, 0.0545 mol) were placed in a double-necked round-bottomed flask, dissolving by ethanol (100 mL) under nitrogen, and the solution was stirred and heated to reflux for 8 h. After the reaction had gone to completion, it was cooled to room temperature and a yellow solid precipitated. The crude product was washed with methanol and filtered under vacuum, followed by drying in a vacuum oven at 70 °C to afford a yellowish powder SBBDA (8.95 g, 61.8%).

^1^H NMR (400MHz, DMSO-d_6_, ppm): δ = 9.601 (s, 2H), δ = 8.683 (s, 2H), δ = 8.041–8.062 (d, 4H), δ = 7.983–8.001 (d, 4H), δ = 7.236–7.253 (d, 4H), δ = 6.792–6.816 (d, 4H), δ = 4.263–4.295 (t, 4H), δ = 1.701–1.732 (m, 4H).

#### 2.3.6. 1,4-Bis{[4-(2,3-epoxypropoxy)phenyliminomethylidene]-benzoyloxyl}butane (SBBDAE)

SBBDA (8.95 g, 0.0167 mol) and benzyltrimethylammonium chloride (0.309 g, 0.001668 mol) were placed in a double-necked round-bottomed flask, adding epichlorohydrin (65.39 mL, 0.834 mol) under nitrogen, and the solution was stirred and heated to reflux for 1 h. Then the solution was cooled to room temperature and a white solid precipitate appeared. The crude product was washed with water and ether, followed by filtration and drying under vacuum at 70 °C to obtain a white powder SBBDAE (8.63 g, 79.7%).

^1^H NMR (400MHz, CDCl_3_, ppm): δ = 8.530 (s, 2H), δ = 8.112–8.132 (d, 4H), δ = 7.942–7.961 (d, 4H), δ = 7.252–7.274 (d, 4H), δ = 6.954–6.983 (d, 4H), δ = 4.443–4.746 (t, 4H), δ = 4.131–4.282 (dd, 2H), δ = 3.962–3.995 (dd, 2H), δ = 3.373–3.383 (m, 2H), δ = 2.916–2.939 (dd, 2H), δ = 2.781–2.792 (dd, 2H), δ = 1.981–2.001 (m, 4H).

#### 2.3.7. 1,8-Bis(4-formylbenzoyloxy)octane (ODA)

1,8-dibromooctane (10 g, 0.0367 mol), 4-formylbenzoic acid (22.23 g, 0.148 mol), K_2_CO_3_ (51.18 g, 0.370 mol) and 18-crown-6 (9.788 g, 0.037 mol) were placed in a double-necked round-bottomed flask, dissolving by DMF (250 mL) under nitrogen, and the solution was stirred and heated to 90 °C for 8 h. After the reaction had gone to completion, the solution was cooled to room temperature, and the K_2_CO_3_ was filtered from the reaction solution. A light coffee-colored clear filtrate was obtained. The filtrate solution was poured into ice water and the white solid was precipitated. The mixture was filtered under vacuum to obtain the white solid. The crude product was dissolved in dichloromethane (DCM) and extracted by 10% potassium hydroxide aqueous solution three times. After the extraction, the organic layer was collected and dried over anhydrous MgSO_4_. The solution was then concentrated and distilled to obtain a white solid, followed by drying in a vacuum oven at 70 °C to afford the white powder ODA (9.43 g, 62.7%).

^1^H NMR(400MHz, CDCl_3_, ppm): δ = 10.101(s, 2H), δ = 8.182–8.202 (d, 4H), δ = 7.941–7.962 (d, 4H), δ = 4.344–4.372 (t, 4H), δ = 1.781–1.792 (m, 4H) δ = 1.422–1.605 (m, 8H).

#### 2.3.8. 1,8-Bis{[4-(4-hydroxy)phenyliminomethylidene]-benzoyloxyl}octane (SBODA)

ODA (9.43 g, 0.0230 mol) and 4-aminophenol (5.014 g, 0.0460 mol) were placed in a double-necked round-bottomed flask, dissolving by ethanol (100 mL) under nitrogen, the solution was stirred and heated to reflux for 8 h. The solution was cooled to room temperature and a yellow solid precipitated. The crude product was washed with methanol, followed by filtration and drying under vacuum at 70 °C to obtain a yellow powder of SBODA (9.02 g, 66.2%).

^1^H NMR (400MHz, DMSO-d_6_, ppm): δ = 9.630 (s, 2H), δ = 8.682 (s, 2H), δ = 8.041–7.982 (q, 8H), δ = 7.234–7.255 (d, 4H), δ = 6.793–6.814 (d, 4H), δ = 4.263–4.292 (t, 4H), δ = 1.702–1.751 (m, 4H), δ = 1.229–1.371 (m, 8H).

#### 2.3.9. 1,8-Bis{[4-(2,3-epoxypropoxy)phenyliminomethylidene]-benzoyloxyl}octane (SBODAE)

SBODA (9.02 g, 0.0152 mol) and benzyltrimethylammonium chloride (0.281 g, 0.00151 mol) were placed in a double-necked round- bottomed flask, adding epichlorohydrin (59.33 mL, 0.756 mol) under nitrogen, and the solution was stirred and heated to reflux for 1 h. The solution was then cooled to room temperature and a white solid precipitate appeared. The crude product was washed with water and ether, followed by filtration and drying under vacuum at 70 °C to afford a white powder SBODAE (8.66 g, 80.9%).

^1^H NMR (400MHz, CDCl_3_, ppm): δ = 8.520 (s, 2H), δ = 8.114–8.131 (d, 4H), δ = 7.934–7.951 (d, 4H), δ = 7.245–7.267 (d, 4H), δ = 6.953–6.975 (d, 4H), δ = 4.333–4.366 (t, 4H), δ = 4.241–4.286 (dd, 2H), δ = 4.013–3.966 (dd, 2H), δ = 3.381–3.389 (m, 2H), δ = 2.916–2.939 (dd, 2H), δ = 2.781–2.790 (dd, 2H), δ = 1.783–1.821(m, 4H), δ = 1.435–1.566 (m, 8H).

### 2.4. Epoxy Sample Preparation

**Figure 2:**
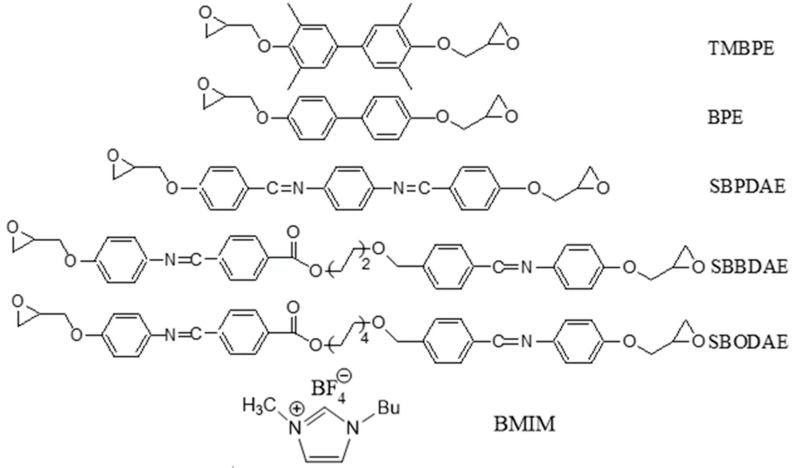
The chemical structures of TMBPE, BPE, SBPDAE, SBBDAE, SBODAE, and BMIM.

The liquid crystal epoxy resins were prepared as follows: (1) mixing the quantitative liquid crystal epoxy (100 g) powders in the right amount of cationic initiator (BMIM, 33 g, Figure 2), and removing the solvent using a vacuum oven; (2) placing the stainless steel mold into the oven and heating to the target temperature; (3) pouring the resin prepared at step 1 into the stainless steel mold, and holding the experiment temperature till the set time was over; (4) after the reaction was finished, cooling down the mold, removing the cured sample of the epoxy resin and measuring the related physical properties.

### 2.5. Epoxy Resin Boron Nitride Sample Preparation

The epoxy resin boron nitride composite was prepared as follows: (1) placing the quantitative liquid crystal epoxy powders and boron nitride into the Vial bottle, closing the cap tightly, and moving the bottle to the LAU disperser machine, setting the working time to be 2 h; (2) taking out the Vial bottle and pouring the mixture into the weighed cationic initiator (BMIM) by a mixer, and removing the solvent using a vacuum oven [33]; (3) putting the epoxy resin filler into the Vial bottle, and using a vacuum mixer to disperse it evenly at 500 rpm for 30 min; (4) after pouring the epoxy resin filling prepared in step 3 into the stainless steel mold, starting to pressurize and heat it to experiment’s target temperature till the set time was over; (5) after the reaction was finished, cooling down the mold, removing the cured sample of the epoxy resin and measuring the related physical properties. The molded specimens were disc-like, with a diameter of 12.5 mm and a thickness of 1 mm.

### 2.6. Measurement of Thermal Conductivity

The thermal conductivity of the molded specimen was measured at 25 °C using TA flashline thermal diffusivity measuring system.

## 3. Results and Discussion

This research is focused on the molecular arrangement of different liquid crystal epoxy resins and the thermal conductivity of their composites. Herein, we synthesized four liquid crystal epoxy resins, BPE, SBPDAE, SBBDAE, and SBODAE; one comparative sample, TMBPE, was purchased from Aaron Chemicals. All the structures were schematized in Figure 2. A cationic curing agent, BMIM, was used in the curing reactions to improve the regularity of the arrangement of epoxy resin molecules [8,33]. This could reduce the phonon scattering during heat conduction and thus increase the thermal conductivity of the resins.

**Figure 3:**
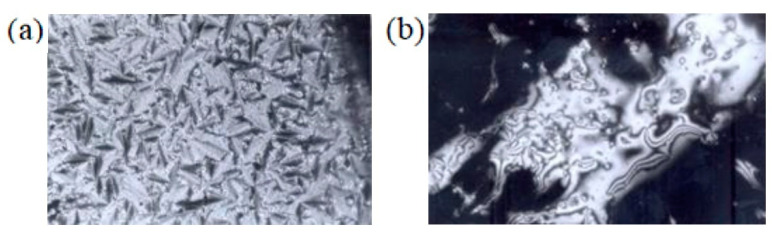
Polarizing optical micrographs (magnification ×320) of SBBDAE in (**a**) smectic A texture obtained from isotropic phase after cooling to 150 °C and (**b**) nematic texture obtained after cooling to 200 °C.

Table 1 lists the thermal transitions and mesomorphic properties of TMBPE and four synthesized LC epoxy monomers. TMBPE shows only crystalline behavior while the other four synthesized LC epoxy monomers reveal mesomorphic behavior. BPE, SBPDAE, and SBODAE reveal the smectic A phase, and SBBDAE exhibits both the smectic A and nematic phases. Figure 3 displays smectic A and nematic textures exhibited by SBBDAE.

**Figure 4:**
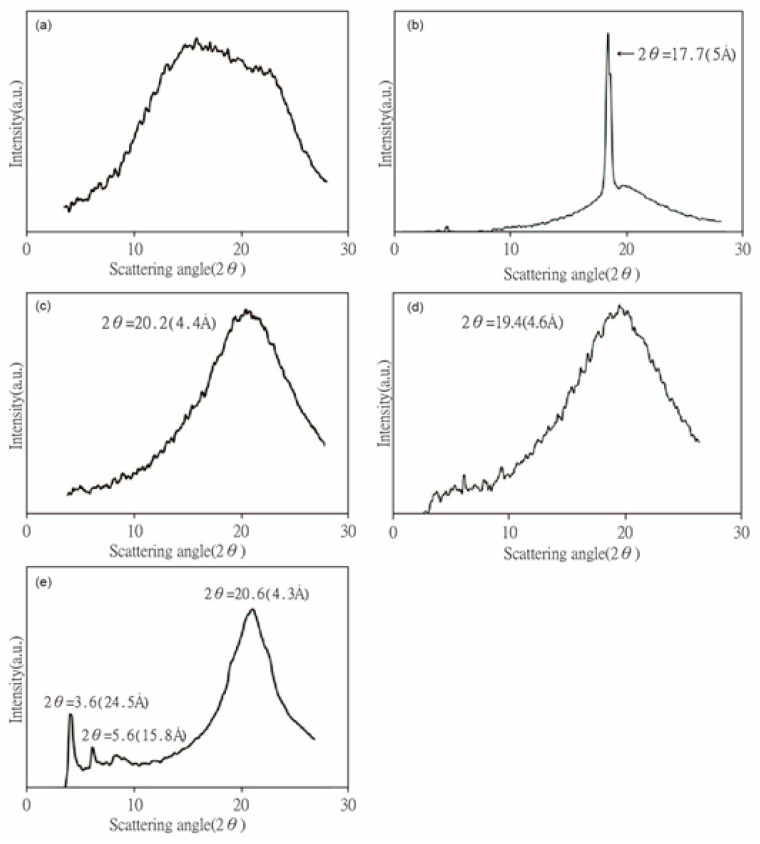
XRD of Thermosets of TMBPE (**a**), BPE (**b**), SBPDAE (**c**), SBBDAE (**d**) and SBODAE (**e**).

The XRD patterns in Figure 4a–e show the regularity of the molecular arrangement in various cured epoxy resins via the cross-linking reaction. TMBPE has a poor crystalline property, and its curing product displays an amorphous phase in Figure 4a. Figure 4b demonstrates that the molecular chain of the cured BPE has a highly regular arrangement structure. It could be contributing to the structure of BPE, which consists of two combined benzene rings. The π-π staking distance was calculated as 5 Å by an angle 2-theta at 17.7 from the XRD pattern. This indicates that a cationic initiator could effectively maintain the arrangement of liquid crystal epoxy molecules during the curing process. Figure 4c–e reveal the high crystallinities of liquid crystal epoxy monomers, SBPDAE, SBBDAE, and SBODAE, and hence their curing products have a moderately regular arrangement structure. However, the BPE curing product still demonstrated a higher regularity than those products. In the chemical structure of SBPDAE, three benzene rings were connected by imine functional groups; the lone pair electron of the nitrogen atom could resonate with the benzene ring to form a longer coplanar surface. This liquid crystal unit has a more complex structure than the BPE, and the rigid structure limited the molecular alignment during the cross-linking reaction. This is a disadvantage for the polymer-chain rearrangement and further suppressed the degree of regularity. The π-π staking distance of its curing product was 4.4 Å. SBBDAE has a double liquid crystal-unit structure; two liquid crystal units were connected by a butyl group. The mobility of the two liquid crystal units would be restricted by each other and make it more difficult to exhibit a regular arrangement that is better than the BPE. The π-π staking distance of its curing product was calculated as 4.6 Å. SBODAE shows a very similar structure to SBBDAE except for the octyl connecting group. The distance between the two liquid crystal units is longer than that of SBBDAE; therefore, the influence by the restriction is lower than it. The SBODAE curing product demonstrated a layered structure, and the staking distance was calculated as 4.3Å. Moreover, the lamellar distances were presented as 15.8 Å and 24.5 Å, respectively.

**Figure 5:**
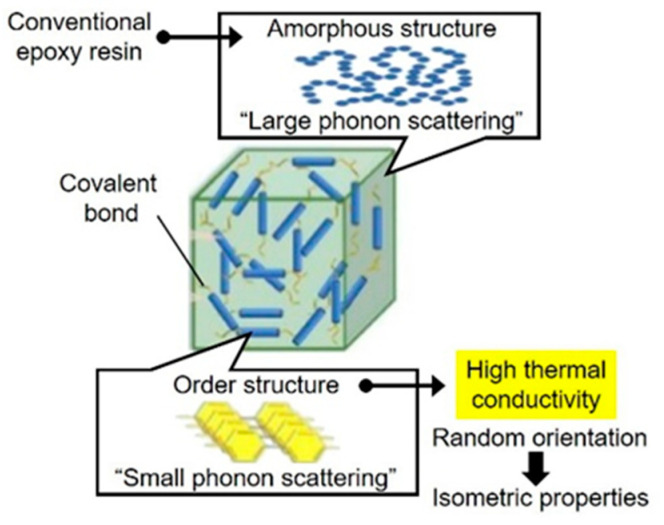
Schematic comparison of the conventional epoxy resin and LC epoxy resin to enhance the thermal conductivity.

Figure 5 shows a comparison of conventional epoxy resin and LC epoxy resin as the matrix to enhance the thermal conductivity of the epoxy/BN composite. The LC epoxy resin induces spontaneous molecular orientation through π–π interactions between the mesogens, thereby improving the crystallinity of the resin. Molecular orientation enables efficient heat transfer through phonon vibration. Therefore the thermal conductivity of LC epoxy resin is much higher than that of conventional epoxy resin. Furthermore, the loading content of the BN can be increased due to the low viscosity of the LC epoxy at the liquid crystalline state.

**Figure 6:**
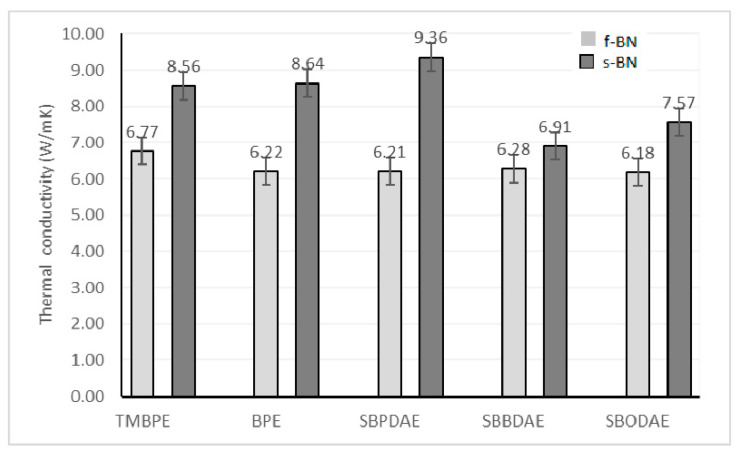
The thermal conductivity of flat boron nitride (f-BN) and spherical boron nitride (s-BN) composites.

To increase the thermal conductivity, different amounts of flat boron nitride (f-BN) and spherical boron nitride (s-BN) were added into the five liquid crystal epoxy resins [18,19,20,21]. The influence of these nano-scaled ceramic materials on the thermal conductivity for liquid crystal epoxy resins is as discussed below. Figure 6 shows the thermal conductivity of flat boron nitride (f-BN) and spherical boron nitride (s-BN) composites.

**Figure 7:**
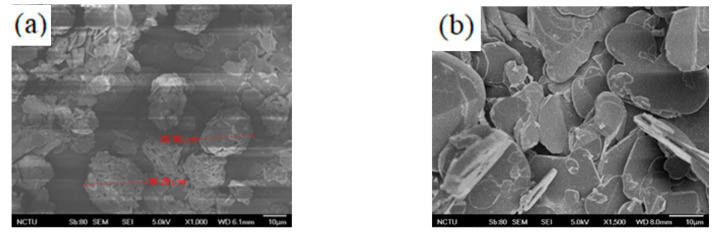
SEM images of fracture surfaces of (**a**) TMBPE/s-BN (60 vol%) (**b**) TMBPE/f-BN (60 vol%).

Herein, all the thermal conductivities of cured LC epoxy composites were measured in the vertical direction of the sample. Due to the two-dimensional structure of the flat boron nitride, it tends to pack horizontally in the cured epoxy composite, leading to a low thermal conductivity in the vertical direction. Therefore, the composites with the f-BN demonstrate an anisotropic behavior, which will lead to many limitations in practical applications. Among these five liquid crystal epoxy resin composites with f-BN, TMBPE shows the highest thermal conductivity (6.77 W/mK), which is much higher than the thermal conductivity of the pure LC epoxies (0.17~0.21 W/mK). Because the molecular chain of TMBPE cured material has a lower degree of regularity, the arrangement of the f-BN makes less of an impact on it than other cured materials. A relatively large proportion of the f-BN could maintain the vertical arrangement, resulting in a higher thermal conductivity from the measurement. On the contrary, the thermal conductivity of the s-BN composite was greater than that of the f-BN composite in the measurement of the vertical direction, as shown in Figure 6. Because of the three-dimensional structure of s-BN, the thermal conductivities in the vertical and horizontal directions show a very similar result. The cured composites with s-BN demonstrate no thermal anisotropy, which means the molecular arrangement of the resins is significantly unaffected by s-BN. Figure 7 shows the SEM images of the fracture surfaces of the TMBPE/s-BN and the TMBPE/f-BN composites. As shown in Figure 7a, s-BN powders are well dispersed in the TMBPE matrix and no isolated s-BN is observed. On the other hand, in Figure 7b, f-BN platelets are clearly observed. The results indicate that the f-BN platelets are arranged in the horizontal direction. This is the reason why TMBPE/s-BN composites reveal higher thermal conductivity than that of the TMBPE/f-BN in the vertical direction.

**Figure 8:**
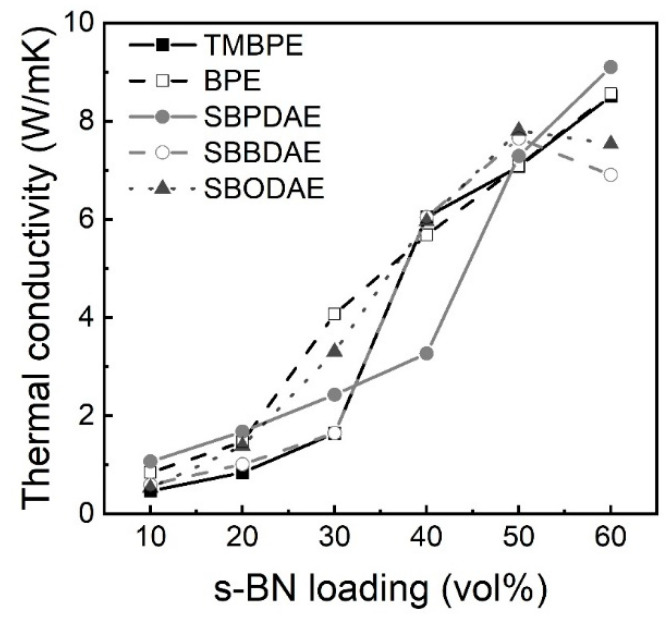
Thermal conductivity of five composites.

Figure 8 depicts the thermal conductivity of five cured liquid crystal epoxy resins with different amounts of boron nitride additive. When 30 vol% of boron nitride was added to liquid crystal epoxy resins, the thermal conductivity of the BPE composite showed the highest result, followed by SBODAE, SBPDAE, SBBDAE, and TMBPE, respectively. The thermal conductivity was highly correlated with the regularity of the resin molecular chain arrangement. When 50 vol% of boron nitride was added to liquid crystal epoxy resins, the thermal conductivity of the SBODAE composite became the highest. This could be attributed to the effect of phonon scattering. It could be seen from the XRD pattern in Figure 4e that the SBODAE cured resin has the lowest π-π staking distance (4.3 Å) and presents a lamellar structure. Therefore, the phonons scattering in the resin would be controlled, leading to the faster transmission speed of it in the resin. When the amount of boron nitride increased to 60 vol%, however, the SBPDAE composite showed the highest (9.36 W/mK) thermal conductivity. The composites of SBBDAE and SBODAE exhibited a significant decrease in the thermal conductivity when the loading percentage of s-BN was over 50 vol%. This could be attributed to the alkyl linkage of both monomers which increases the soft domain of the polymer arrangement. This soft domain in the polymer-chain orientations could cause the aggregation of the s-BN filler when its loading percentage increases, leading to a decreasing thermal conductivity. In the case of BPE, its thermal conductivity was nearly proportional to the loading percentage of s-BN. This may be due to its high crystallinity, and thus two different thermal conducting routes from polymer domain and ceramic filler were formed individually. In addition, TMBPE showed an amorphous behavior (Figure 4a), and its thermal conductivity raised only after 30 vol% of s-BN was introduced. This may be caused by the fillers contacting each other to form a new heat transport network after a particular amount of insertion.

**Figure 9:**
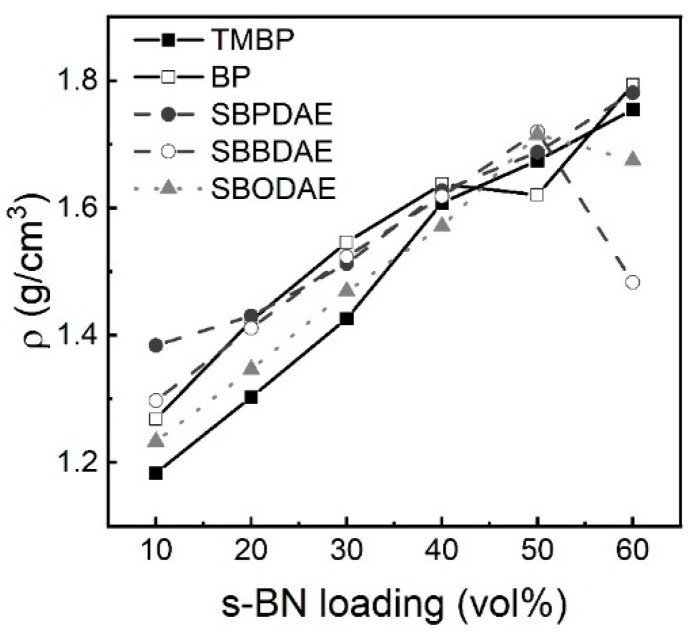
Density of five liquid crystal epoxy composites with different amounts of boron nitride additive.

Figure 9 shows the density of five liquid crystal epoxy composites with different amounts of boron nitride additive. Generally, the more boron nitride is added, the higher density of the liquid crystal epoxy composite will be observed. However, when the addition amount of boron nitride was over 50 vol%, it exhibited a decrease in the density of composites SBBDAE and SBODAE. This is consistent with the above-mentioned results of the thermal conductive trend; the soft alkyl linkages of these two monomer structures lead to the aggregation of s-BN. Therefore, the cavity was formed between the interface of polymer blocks and ceramic filler, which causes a significant decrease in the density. On the other hand, BPE-cured resin is the most crystalline resin, and it would not be easy to stack with boron nitride. Two individual domains of the organic and inorganic portion may be formed to build their own heat transport network, and thus their thermal conductivity demonstrated a non-monotonic increasing trend with an increasing concentration of the filler.

In summary, choosing an appropriate polymer structure is important for getting a high thermally conductive material. Polymers with high crystallinity can lead to high thermal conductivity. The thermal conductivity of polymer resins can also be enhanced by adding high thermal conductive fillers, such as boron nitride. A 3D filler of s-BN is better than the 2D f-BN because of the dimensional orientation. Moreover, the filler distribution also plays an important role in determining the thermal conductivity of the composite resin. The filler aggregation could suppress the thermal conductivity enhancement, especially in high filling concentrations. The composites of SBBDAE and SBODAE are a good example of this phenomenon, while the alkyl linkages are designed for the improvement of its solubility. SBPDAE, which has a moderate inter-chain interaction in its polymer arrangement, showed the best performance of thermal conductivity in this study.

## 4. Conclusions

In this study, BPE, SBPDAE, SBBDAE, and SBODAE liquid crystal epoxy resins were successfully synthesized. Curing reactions were carried out with a cationic curing agent (BMIM), which could improve the regularity of the arrangement of epoxy resin molecules. The order of the molecular arrangement rules measured by X-ray was BPE, SBODAE, SBPDAE, SBBDAE, and TMBPE, respectively. The thermal conductivity of the liquid crystal epoxy resin composites is larger than that of the liquid crystal epoxy resins. The thermal conductivity of the spherical boron nitride composite is greater than that of the flat boron nitride composite. Generally, the more boron nitride was added, the higher density and thermal conductivity of the liquid crystal epoxy composite were observed. In addition, the polymer-chain morphology and the filler distribution play a notable role in the enhancement of their thermal conductivity.

## Figures and Tables

**Table 1:** Thermal transitions of compounds TMBPE, BPE.

SBPDAE, SBBDAE and SBODAE	
liquid crystal epoxy	Phase transitions
**TMBPE**	K 105.2 I
**BPE**	K 125.2 S_A_ 158.3 I
**SBPDAE**	K 185.5 S_A_ 262.9 I
**SBBDAE**	K 129.3 S_A_ 173.8 N 258.6 I
**SBODAE**	K 142.3 S_A_ 161.7 I
K = crystal, S_A_ = smectic A, N = nematic, I = isotropic (a) determined by optical polarizing microscopic observation.	

## Data Availability

The data used to support the findings of this study are included in the article.
